# Convergence of genetic and environmental factors on parvalbumin-positive interneurons in schizophrenia

**DOI:** 10.3389/fnbeh.2013.00116

**Published:** 2013-09-03

**Authors:** Zhihong Jiang, Rita M. Cowell, Kazu Nakazawa

**Affiliations:** Unit on Genetics of Cognition and Behavior, National Institute of Mental Health, NIHBethesda, MD, USA; Department of Psychiatry and Behavioral Neurobiology, University of Alabama at BirminghamBirmingham, AL, USA

**Keywords:** animal model, fast-spiking neurons, GABA neuron, NMDA receptors, oxidative stress, PGC-1*α*, social isolation

## Abstract

Schizophrenia etiology is thought to involve an interaction between genetic and environmental factors during postnatal brain development. However, there is a fundamental gap in our understanding of the molecular mechanisms by which environmental factors interact with genetic susceptibility to trigger symptom onset and disease progression. In this review, we summarize the most recent findings implicating oxidative stress as one mechanism by which environmental insults, especially early life social stress, impact the development of schizophrenia. Based on a review of the literature and the results of our own animal model, we suggest that environmental stressors such as social isolation render parvalbumin-positive interneurons (PVIs) vulnerable to oxidative stress. We previously reported that social isolation stress exacerbates many of the schizophrenia-like phenotypes seen in a conditional genetic mouse model in which NMDA receptors (NMDARs) are selectively ablated in half of cortical and hippocampal interneurons during early postnatal development (Belforte et al., 2010). We have since revealed that this social isolation-induced effect is caused by impairments in the antioxidant defense capacity in the PVIs in which NMDARs are ablated. We propose that this effect is mediated by the down-regulation of PGC-1*α*, a master regulator of mitochondrial energy metabolism and anti-oxidant defense, following the deletion of NMDARs (Jiang et al., 2013). Other potential molecular mechanisms underlying redox dysfunction upon gene and environmental interaction will be discussed, with a focus on the unique properties of PVIs.

## Introduction

Schizophrenia is a chronic and complex neuropsychiatric disorder that affects approximately 1% of the population worldwide (Insel, 2010). The characteristic features of schizophrenia have been categorized into three major symptom domains (DSM-IV, American Psychiatric Association, 1994). Positive symptoms are psychotic behaviors not seen in healthy people, such as hallucillations, delusions, disorganized thinking, and movement disorders which, in part, can be relieved through the use of current antipsychotic medications. Negative symptoms are associated with disruption of normal emotions and behaviors, such as motivational impairment (avolition or amotivation), affective dysregulation (depression, mania), and social withdrawal. Cognitive symptoms include poor executive functioning, trouble focusing or paying attention, and problems with working memory. The onset of symptoms usually occurs during late adolescence and early adulthood.

Based on classical family, twin and adoption studies, genetic theories of schizophrenia etiology have been prominent since 1960s (Gottesman and Shields, 1976; Cardno and Gottesman, 2000; Carter et al., 2002; Sullivan et al., 2003; Tienari et al., 2004; Lichtenstein et al., 2009). Early linkage studies produced the first list of gene candidates, and a large body of gene association work identified some of these candidate genes as true susceptibility factors (Owen et al., 2005; Ross et al., 2006; Straub and Weinberger, 2006). Recently, analysis of data generated using genome-wide mass-marker technology (such as genome-wide association studies (GWAS) and next generation sequencing) has raised the possibility that thousands of common polymorphisms (Purcell et al., 2009) and multiple rare copy-number variations (CNVs) in several different genomic areas contribute to schizophrenia risk (Stefansson et al., 2008; Stone et al., 2008; Walsh et al., 2008; Xu et al., 2008; McCarthy et al., 2009; Ingason et al., 2011). Although GWAS from the psychiatric genomics consortium (PGC), a collaboration across more than 300 scientists in 80 institutions and 20 countries, have been carried out to investigate a large number of polymorphisms from thousands of patients (61,220 subjects, Smoller et al., 2013), no single gene or allele has independently been identified as a key risk factor in a large number of people.

One plausible explanation for the weak association of schizophrenia with an individual gene is that genetic risk is critically dependent on environmental context. A considerable body of evidence suggests that an interaction between genetic vulnerability and environmental factors (GxE) leads to the manifestation of schizophrenia. Even monozygotic twins who share identical genes have a concordance rate of 41–65% (Cardno and Gottesman, 2000). Epidemiologic studies suggest that a diversity of factors, including prenatal infection/immune activation, paternal age, malnutrition, hypoxia-related obstetric complications, and childhood/adolescence social stress and cannabis abuse, are associated with an increased risk for the development of this disorder (see reviews, Barkus and Murray, 2010; van Os et al., 2010; Brown, 2011; Meyer, 2013).

Various theories have been proposed to explain how genes and the environment interact to give rise to complex neuropsychiatric disorders. The “Diathesis-stress” model of schizophrenia, introduced by Rosenthal D. and Bleuler M. in 1963 (Ingram and Luxton, 2005) and recently revisited by Fowles (1992), posits that development of schizophrenia requires both biological vulnerability (diathesis) and stressful life. Weinberger (1987) went on to propose a similar model in the context of brain development and maturation, suggesting that an environmental stressor during early life brain maturation is necessary to trigger the onset of full-blown psychotic behavior. In the framework of the neurodevelopmental theory, a “two-hit” model was proposed and two critical time windows associated with brain early development and maturation during adolescence were identified as sensitive periods for exposure to environmental insults that could account for the emergence of symptoms of schizophrenia (Bayer et al., 1999; Keshavan, 1999).

However, there is a fundamental gap in our understanding of the molecular mechanisms by which environmental factors interact with genetic susceptibility during brain development to trigger psychosis. A large number of neurodevelopmental animal models have been developed to investigate the biological basis for GxE interactions and to evaluate novel pharmacological therapies for their treatment (review, Meyer and Feldon, 2010). For example, inflammatory responses after infection and cytokine-mediated effects on brain development have been studied using polyinosinic:polycytidylic acid (poly I:C) and lipopolysaccharide (LPS) in rodents; protein deprivation and vitamin D deficiency to mimic malnutrition; perinatal/postnatal hypoxia models for obstetric complications; maternal isolation, post-weaning social isolation/chronic restraint stress have been used to study psychosocial stress effects on brain development. Genetic manipulation of schizophrenia susceptibility genes in rodents, such as DISC1, NRG1/ErbB4, and COMT has been largely explored as well.

Interestingly, one of the common findings in both animal models and postmortem tissue from patients with schizophrenia is a reduction of the calcium buffer parvalbumin (PV) mRNA or protein level in cortical fast-spiking (FS) interneurons. PV-positive interneurons (PVIs), which account for ~40% of the total cortical Gamma-amminobutyric acid (GABA) ergic interneurons in rodents (Rudy et al., 2011) and 25% in primates (Condé et al., 1994) and include basket and chandelier cells, represent a unique class of interneurons. A significant mRNA reduction of PV (Hashimoto et al., 2003, 2008; Mellios et al., 2009; Fung et al., 2010; Volk et al., 2012), GAD67 (Hashimoto et al., 2003; Curley et al., 2011) or GAT1 (Volk et al., 2012) has been found in PVIs in the dorsolateral prefrontal cortex of individuals with schizophrenia. Altered GABA neurotransmission at the synapse between PV-positive chandelier neurons and the pyramidal cell axon initial segment (AIS), such as decreased density of chandelier neuron axon cartridges immunoreactive for GAT-1 (Woo et al., 1998; Pierri et al., 1999), increased GABA_A_ receptor *α*2 subunit at AIS of pyramidal neurons (Volk et al., 2002) and increased GABA_A_ receptor binding (Benes et al., 1992, 1996), was also found in schizophrenic patients. Furthermore, factors required for development of PVI including the transcription factor Lhx6 and brain derived neurotrophic factor (BDNF) and its receptors are reduced in a subset of individuals with schizophrenia (Cobos et al., 2006; Liodis et al., 2007; Zhao et al., 2008, see reviews, Huang et al., 1999; Weickert et al., 2003; Hashimoto et al., 2005; Weickert et al., 2005; Woo and Lu, 2006; Volk et al., 2012).

Dysfunction of PVIs has been indicated in many neurodevelopmental animal models as well. Studies in reverse-translational models using schizophrenia-risk genes, such as ErbB4 (Fisahn et al., 2009; Neddens and Buonanno, 2010), DISC1 (Hikida et al., 2007; Shen et al., 2008; Ayhan et al., 2011), DTNP1 (Ji et al., 2009; Carlson et al., 2011), BDNF (Sakata et al., 2009) and glutamate cysteine ligase modifier (GCLM; Steullet et al., 2010; Cabungcal et al., 2013a), consistently observed a decreased number or impaired function of PVIs in the hippocampus or cortex. Also, intensity of PV immunoreactivity and/or loss of PV-positive cells has been reported in the hippocampus and cortex of mice with developmental inflammation [prenatal poly I:C injection (Meyer et al., 2008; Ducharme et al., 2012; Piontkewitz et al., 2012); neonatal LPS injection (Jenkins et al., 2009)], exposure to hypoxia (Gerstein et al., 2005; Fagel et al., 2009), chronic social isolation stress (Harte et al., 2007; Schiavone et al., 2009, 2012; Filipovic et al., 2013), ventral hippocampal lesions (Tseng et al., 2008; François et al., 2009) or prenatal (E17) methylazoxymethanol acetate (MAM) injections (Lodge et al., 2009; Gastambide et al., 2012). Pharmacological manipulation by NMDA receptor (NMDAR) antagonists, such as repetitive injection of ketamine (Behrens et al., 2007), prenatal exposure to MK-801 (Abekawa et al., 2007) or perinatal phencyclidine (PCP) injection (Wang et al., 2008), all resulted in a decrease in the number of cortical PVIs. These data suggest that PV immunoreactivity and PVI cells themselves are particularly sensitive to neurodevelopmental insults.

PVI cell loss and dysfunction have serious consequences for cortical and hippocampal function. PVIs produce sustained, high-frequency trains of brief action potentials with large and fast after-hyperpolarization and little spike frequency adaptation. They have the lowest input resistance and the fastest membrane time constant among all interneurons, features that ensure fast synaptic responses (Connors and Gutnick, 1990; Markram et al., 2004; Ascoli et al., 2008; Goldberg et al., 2008). FS PVIs are interconnected via chemical and electrical synapses (Galarreta and Hestrin, 1999; Gibson et al., 1999) and have a highly divergent synaptic output to principle neurons (Gulyás et al., 1999). Inhibitory synapses between PVIs synchronize action potential activity within basket cell network, whereas inhibitory synapses between basket cells and principle neurons distribute this synchronized activity to the principle neuron population. Accordingly, multiple lines of evidence indicate that FS PVIs are essential for the generation of gamma oscillations (Csicsvari et al., 2003; Hájos et al., 2004; Mann et al., 2005; Cardin et al., 2009; Sohal et al., 2009), which provides a temporal structure for information processing and contributes to cognitive functions including attention (Fries et al., 2001, 2008; Siegel et al., 2008), perception (Rodriguez et al., 1999) and working memory (Howard et al., 2003). Cognitive impairments such as deficits in working memory, attention, and executive function are particularly evident in patients with schizophrenia (Elvevåg and Goldberg, 2000), and abnormalities in gamma oscillations may contribute to these deficits. Schizophrenic patients exhibit decreases in the power or synchrony of gamma oscillations during responses to sensory stimulation or cognitive tasks (Gallinat et al., 2004; Spencer et al., 2004; Symond et al., 2005; Wynn et al., 2005; Cho et al., 2006; Ford et al., 2007, 2008; Haenschel et al., 2009; Uhlhaas and Singer, 2010). Thus, abnormalities of FS PVIs may underlie the cognitive disturbances associated with schizophrenia.

Considering the substantial evidence for interneuron dysfunction and NMDAR hypofunction in schizophrenia, we investigated the impact of NMDAR deletion specifically from interneurons using a Cre/*loxP* system in which early postnatal ablation is restricted to 40–50% of the cortical and hippocampal interneurons, with the majority of cre-targeted cells being PV-positive [NMDAR (GluN1) knockout mouse strain (Ppp1r2-cre/fGluN1 KO mice; Belforte et al., 2010)]. In this mouse, NMDARs were functionally eliminated in the early postnatal period (Cre recombination was detectable in the cortex and hippocampus firstly at postnatal day seven and almost completed by postnatal three weeks). Reduced GAD67 and PV protein levels and reduced GABA release were observed from GluN1-depleted interneurons in mutant animals, and mutant mice exhibited cortical disinhibition evidenced by increased firing of cortical excitatory neurons and reduced neuronal synchrony. At the behavioral level, this mutant mouse reproduced positive, negative, cognitive and anxiety-like behavioral phenotypes that resemble the symptoms of human schizophrenia. Most mutant behavioral phenotypes were first observed >12 weeks of age, suggesting a latency period between GluN1 knockout and the emergence of these phenotypes (Belforte et al., 2010; Nakazawa et al., 2012). Interestingly, social isolation initiated during adolescence exacerbated the expression of these phenotypes in the mutant (Jiang et al., 2013).

Importantly, schizophrenia-like pathophysiological and behavioral phenotypes were not observed when genetic GluN1 ablation in the same subpopulations of GABAergic (gamma-aminobutyric acid)

 neurons occurred after adolescence (Belforte et al., 2010), suggesting that GluN1 deletion is most detrimental during the postnatal maturation of PVIs. Furthermore, a prominent increase of oxidative stress was observed in KO mice, particularly in cortical PVIs, with post-weaning social isolation sharply exacerbating redox dysfunction. Chronic treatment with apocynin (APO), an antioxidant and reactive oxygen species (ROS) scavenger, abolished oxidative stress signs and partially alleviated schizophrenia-like behavioral phenotypes in KO mice (Jiang et al., 2013).

**Figure 1:**
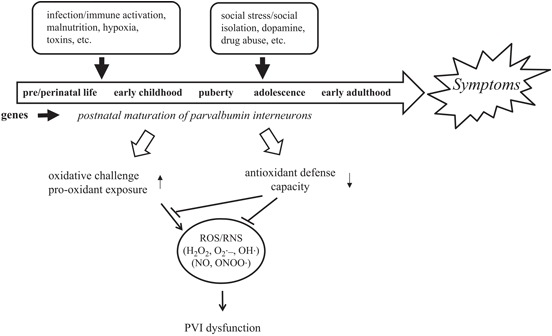
**PVIs are vulnerable to oxidative stress induced by GxE during development.** An interaction between genetic susceptibility and environmental insults disrupts normal brain development and leads to the manifestation of schizophrenia. Unique properties of PVIs make them vulnerable to redox imbalance induced by GxE.

In the context of this new data and the substantial evidence for PVI dysfunction in schizophrenia, we propose that social isolation in development exacerbates schizophrenia-like phenotypes via cortical oxidative stress in PVIs (Jiang et al., 2013). These data are in line with the “diathesis–stress” and neurodevelopmental theories for the etiology of schizophrenia and suggest that oxidative stress is one of central factors linking genetic and environmental risks to GABAergic dysfunction (Figure 1). Below, we discuss in detail the evidence for the involvement of oxidative stress in the pathophysiology of schizophrenia, the specific properties of PVIs that render them vulnerable to oxidative stress, and a potential molecular pathways which could account for environmentally-induced oxidative stress in PVIs.

## Oxidative stress as a convergence point for genetic and environmental susceptibility to schizophrenia

Growing body of evidence suggests that oxidative stress plays a significant role in the pathogenesis of schizophrenia (see reviews, Behrens and Sejnowski, 2009; Do et al., 2009; Yao and Keshavan, 2011). Oxidative stress occurs when ROS or reactive nitrogen species (RNS) are over-produced or antioxidant defense mechanisms fail to counterbalance endogenouse ROS/RNS generated from normal oxidative metabolism or from pro-oxidant environmental exposure. Excessive ROS or RNS can lead to DNA damage and membrane damage due to lipid peroxidation and protein dysfunction. Mammalian antioxidant defense system include ROS detoxifying enzymes such as superoxide dismutases (SOD), catalase (CAT) and glutathione peroxidase (Gpx), and nonenzymatic antioxidant components such as albumin, uric acid, bilirubin, glutathione (GSH), ascorbic acid (vitamin C) and a-tocopherol (vitamin E). Reduced levels of ROS detoxifying enzymes and antioxidants have been reported in schizophrenia (Suboticanec et al., 1990; Reddy et al., 1991; McCreadie et al., 1995; Mukerjee et al., 1996; Yao et al., 1998, 2000; Do et al., 2000; Ranjekar et al., 2003; Reddy et al., 2003; Yao et al., 2006; Dadheech et al., 2008; Raffa et al., 2009; Gawryluk et al., 2011; Coughlin et al., 2013), in addition to increased levels of lipid peroxides in blood (Zhang et al., 2006; Al-Chalabi et al., 2009; Padurariu et al., 2010), platelets (Dietrich-Muszalska et al., 2005), plasma (Mahadik et al., 1998) and urine (Dietrich-Muszalska and Olas, 2009a). Increased protein modification has been also demonstrated in plasma (Dietrich-Muszalska et al., 2009), platelets (Dietrich-Muszalska and Olas, 2009b) and postmortem brains (Wang et al., 2009).

### Genetic origin of oxidative stress

Interestingly, a number of genes involved in antioxidant defense have been linked to susceptibility for schizophrenia. These include MnSOD (Akyol et al., 2005), glutathione S-transferases (Saadat et al., 2007; Nafissi et al., 2011), and a subunit of the key GSH-synthesizing enzyme GCLM (Tosic et al., 2006; Gysin et al., 2007, 2011). A mitochondrial DNA sequence variation affecting a subunit of NADPH-ubiquinone reductase (complex I), a component of the electron transport chain responsible for generating superoxide, has also been associated with schizophrenic patients and with increased superoxide levels in postmortem brain samples (Marchbanks et al., 2003). Furthermore, abnormal proteins encoded by schizophrenia susceptibility genes (PRODH, DISC1, DAOA, NRG1, G72) cause oxidative stress and/or hypersensitivity to oxidative stress (Goldshmit et al., 2001; Krishnan et al., 2008; Drews et al., 2012) or mitochondrial dysfunction (Park et al., 2010).

### Oxidative stress induced by environmental insults

In addition to our own evidence for social isolation-induced oxidative stress, other studies have demonstrated links between environmental stressors and oxidative stress. ROS elevation was found after restraint stress (Madrigal et al., 2001; Chakraborti et al., 2007; Sahin and Gumuslu, 2007), and social isolation rearing in rats induces behavioral changes akin to schizophrenia, mediated by an oxidative stress following NADPH oxidase (Nox-2) elevation in pyramidal neurons (Schiavone et al., 2009, 2012) or associated with decreased superoxide dismutase activity and oxidized/reduced glutathione ratio in the cortico-striatal area (Möller et al., 2011). As in our model, APO treatment alleviated the signs of oxidative stress and behavioral deficits following social isolation (Schiavone et al., 2009, 2012). Furthermore, oxidative stress has been speculated to be an important downstream mechanism of inflammation-mediated immune responses. Maternal immune activation by LPS in rodents triggers oxidative stress (Lanté et al., 2007; Kaneko et al., 2012; Oskvig et al., 2012), and maternal N-acetyl cysteine (NAC, a glutathione precursor) treatment prevents LPS-induced adverse developmental outcomes including elevation of cytokines in maternal and fetal compartments (Xu et al., 2005; Beloosesky et al., 2006), fetal death, preterm labor (Buhimschi et al., 2003), hypomyelination (Paintlia et al., 2004), and impairments in spatial memory and hippocampal long-term potentiation in the offspring (Lanté et al., 2007). Protein malnutrition (Feoli et al., 2006a,b) and hypoxia (Dafre et al., 2003; Sheldon et al., 2008; Niatsetskaya et al., 2012) can also increase oxidative stress.

### GxE-induced oxidative stress

A number of studies have investigated whether prenatal and/or postnatal environmental insults interact with specific genetic factors to cause oxidative stress and increase the risk of long-lasting neurodevelopmental brain dysfunction. Using mice which exihibit a 60–70% reduction in GSH due to ablation of the GCLM gene, Kim Q. Do and her colleagues have shown that impaired GSH synthesis is associated with morphological, neurochemical anomalies and behavioral phenotypes similar to those in schizophrenia patients (Steullet et al., 2010; Kulak et al., 2012). They also found elevated oxidative stress indicated by increased levels of 8-oxo-7, 8-dihydro-2′-deoxyguanosine (8-OH-dG), a marker of oxidized DNA, in ventral hippocampus and anterior cingulate cortex (Steullet et al., 2010; Cabungcal et al., 2013a). An exogenous dopamine stress induced by a dopamine reuptake inhibitor, GBR-12909, led to exacerbated 8-OH-dG labeling in the anterior cingulate cortex of GCLM KO mice but not wild-type mice. Treatment with NAC fully prevented the increase in 8-OH-dG labeling induced by GBR-12909 in KO mice (Cabungcal et al., 2013a).

In light of our recent study indicating an interaction between NMDAR hypofunction and oxidative stress (Jiang et al., 2013), it is important to note that the relationship of NMDAR hypofunction to oxidative stress was initially observed in the rodent models treated with NMDAR antagonists. Chronic perinatal PCP administration reduced glutathione levels and produced long-term alteration of antioxidant defense in the corticolimbic areas of the rat brain (Radonjic et al., 2010). Similarly, repetitive exposure to ketamine, another NMDAR antagonist, activated the superoxide-producing enzyme Nox-2 in mouse brains (Behrens et al., 2007; Zhang et al., 2008). Elevation of cortical ROS level was also found in our Ppp1r2-cre/fGluN1 KO mice when NMDAR was eliminated in cortical PVIs during early postnatal period. Interestingly, post-weaning social isolation sharply exacerbated ROS level in KO mice. Chronic treatment with APO abolished oxidative stress signs and partially alleviated schizophrenia-like behavioral phenotypes in KO mice. These indicated that oxidative stress does contribute to behavioral impairments of this mutant line and social isolation exacerbated schizophrenia-like phenotypes via cortical oxidative stress (Jiang et al., 2013).

## PV interneuron-specific vulnerability to oxidative stress

It has been observed in several lines of animal studies that PVIs are vulnerable to oxidative stress during development. Behrens et al. (2007, 2008) found that repetitive adult exposure to the NMDAR antagonist ketamine increases the levels of the proinflammatory cytokine interleukin-6 (IL-6) in brain which, through activation of the superoxide-producing enzyme Nox-2, leads to the loss of the GABAergic phenotype of PVIs. Pretreatment of animals with APO, or with the SOD-mimetic C3 reduces superoxide production and prevents the loss of PV immunoreactivity (IR) induced by ketamine. Nox-2 deficiency completely prevented the loss of PVIs in the prelimbic regions (Behrens et al., 2008). The reversible effects in adult exposure (Behrens et al., 2008) were not observed for the animals with perinatal exposure of ketamine, suggesting that perturbation of the excitatory/inhibitory balance during early life produces oxidative stress that has profound effect on PVI maturation. In fact, exposure of wild type mice to ketamine on postnatal day 7, 9, and 11 is sufficient to cause a reduction in PV-IR in adulthood without death of interneurons (Powell et al., 2012), suggesting that blockade of NMDAR signaling is sufficient to alter the expression profile of FS interneurons.

The idea that PVIs are more susceptible to oxidative stress during development is also supported by the evidence that early postnatal PCP injection can selectively reduce PVI numbers (Wang et al., 2008; Nakatani-Pawlak et al., 2009) and cause redox dysregulation (Radonjic et al., 2010) in corticolimbic areas. Cabungcal et al. (2007) showed that a transient brain GSH deficit induced by BSO (Lbuthionine-(S, R)-sulfoximine) during early postnatal period is sufficient to cause cognitive impairment as well as decreased numbers of PVIs in adulthood. Using GCLM KO mice, Cabungcal et al. (2013a) demonstrated that impaired synthesis of glutathione delays maturation of PVIs in the anterior cingulate cortex as indicated by reduced PVI numbers, but not calretinin and calbidin positive interneuron numbers, and a reduction in the density of perineuronal nets (PNNs), specialized extracellular matrix components concentrated around PVIs. These effects of reduced glutathione synthesis were only evident during early development but not in later stages. More interestingly, they found that an additional oxidative challenge induced by the dopamine reuptake inhibitor GBR-12909 in preweaning or pubertal but not in young adult GCLM KO mice reduces the number of PVIs in anterior cingulate cortex. Additionally, PVIs in rats with a neonatal central hippocampal lesion exhibit oxidative stress prior to symptom onset as indicated by increased 8-OH-dG marker intensity in most PVIs in the prefrontal cortex (O’Donnell, 2012). Treatment with the GSH precursor NAC during juvenile and adolescent periods reverses the loss of PVIs and restores deficits in prepulse inhibition (O’Donnell, 2012). Collectively, these data suggest that PVIs are vulnerable to oxidative stress, particularly during their development. In our Ppp1r2-cre/fGluN1 KO mice, we observed the reduction of PV-IR and oxidative stress increase on cortical PVIs, not calretinin or calbidin positive interneurons, following the post-weaning social isolation. Reduction of PV-IR upon social isolation was prevented by chronic treatment of APO, suggesting that reduced PV protein level is associated with elevated oxidative stress in PVIs (Jiang et al., 2013).

### Postnatal development and maturation of fast-spiking PVIs

Specific vulnerability of PVIs to developmental stressors that cause oxidative stress could result from their relatively late time course of maturation. The mammalian GABAergic system develops slowly during early postnatal life; in the neonatal brain, GABA is the principal excitatory transmitter that induces depolarization and that are important for the early development of the neural network (Ben-Ari et al., 2004). Conversion of GABAergic transmission from depolarizing to hyperpolarizing during the first postnatal week in rodents depends on the expression of the K+/Cl- cotransporter (KCC2) and the Cl- electrochemical potential (Ben-Ari et al., 2004, 2012). GABA signaling not only regulates interneuron axon branching and synapse formation during the maturation of inhibitory innervations (Chattopadhyaya et al., 2007) but also regulate synapse elimination and axon pruning (Wu et al., 2012).

Studies on multiple mammalian species have shown that cortical FS PVIs develop dramatically during early postnatal life. In rodents, transcriptional and electrophysiological maturation of neocortical FS interneurons from slow (low gamma band frequency) into fast (upper gamma band) signaling devices occurs over the first four weeks (Doischer et al., 2008; Okaty et al., 2009; Goldberg et al., 2011). PVIs start to express parvalbumin mRNA and protein by postnatal first week (de Lecea et al., 1995; Itami et al., 2007). The electrochemical properties of PVIs change around the same developmental age; the K+ channel subunits Kv3.1 and Kv3.2, sodium channels Scn8a and Scn4b, and two-pore K+ leak channels TWIK1 and TASK1 are developmentally regulated and contribute to the establishment of the FS behavior characteristics of PVIs (Du et al., 1996; Rudy and McBain, 2001; Tansey et al., 2002; Grieco and Raman, 2004; Grieco et al., 2005; Levin et al., 2006; Okaty et al., 2009; Goldberg et al., 2011). It was also found that the synaptogenesis of both electrical and GABAergic connections among FS PVIs and PVI-pyramidal neuron microcircuit in rodent was not established until the second postnatal week (Pangratz-Fuehrer and Hestrin, 2011; Yang et al., 2012), and maturation of perisomatic innervations by PVIs is activity and experience dependent (Chattopadhyaya et al., 2004; Jiao et al., 2006). Mature PVIs are wrapped by PNNs, and removal of PNNs increased the excitability of interneurons (Dityatev et al., 2007). Recently it was revealed that PNNs protected mature PVIs against oxidative stress and excessive ROS challenge during early life disrupted PNNs maturation around PVIs and made them more susceptible to oxidative stress (Cabungcal et al., 2013b).

A critical period for PVI maturation is adolescence. Studies in rodents, primates and human subjects indicate that expression of PV protein or mRNA and the density of PVIs processes increase during the pre-pubertal period, peaking during adolescence (Anderson et al., 1995; Erickson and Lewis, 2002; Fung et al., 2010; Caballero et al., 2013). FS PVIs in PFC undergo dramatic changes in the composition of glutamatergic receptors during adolescence: the NMDA/AMPA ratio is dramatically decreased in adolescence, but returns to juvenile levels in adults (Wang and Gao, 2009) and more FS PVIs express calcium permeable AMPA receptors during adolescence (Wang and Gao, 2010). Dopamine modulation of FS PVIs changes dramatically during adolescence (Tseng and O’Donnell, 2007; O’Donnell, 2010, review). Interestingly, in a human study, Uhlhaas et al. (2009) found that cortical networks undergo a transient destabilization from late adolescence to early adulthood, reflected by significant reductions of phase synchrony and induced gamma-band power.

### The high energy demand of PVIs

The majority of PVIs are FS (86%, Pawelzik et al., 2002) and PVIs receive extremely dense excitatory innervations (Gulyás et al., 1999), suggesting that PVIs are often in a depolarized state. As a consequence of intense neuronal firing, the constant requirement for maintenance of sodium and potassium balance puts a high demand on cellular energetics. Consistent with this idea, PVIs have particularly larger numbers of mitochondria and higher cytochrome c content than other neurons (Gulyás et al., 2006). Also, it was recently reported that gamma oscillations are also especially energy demanding and require both high level of complex I and strong functional performance of mitochondria. Kann et al. (2011) found that the amount of oxygen consumption of gamma oscillations reaches that of seizure-like events, and gamma oscillations utilize mitochondrial oxidative capacity near limit. Groups of studies indicated that gamma oscillations are exquisitely sensitive to mitochondrial dysfunction (Fano et al., 2007; Huchzermeyer et al., 2008; Hájos et al., 2009; Pietersen et al., 2009; Whittaker et al., 2011) under conditions with a variety of mitochondrial respiratory chain inhibitors through an effect on FS interneurons. Interestingly, Kann et al. (2011) found that gamma oscillation power, oxygen consumption and complex I subunits are particularly higher in hippocampal subfield CA3. Kim Q. Do and her colleagues observed a selective increase of oxidative stress and a decrease of PV-IR interneurons in ventral CA3/DG in GCLM KO mice with a deficit of antioxidant capacity in the brain (Steullet et al., 2010). This may be related to particularly high energy demanding in these areas.

In line with a high metabolic rate of PVIs, these neurons (and transcription of PV itself) are especially vulnerable to stimuli that compromise mitochondrial function. For example, PVIs in the striatum are sensitive to 6-hydroxydopamine (Salin et al., 2009), quinolic acid (Giampà et al., 2006), rotenone (Lapointe et al., 2004), and methamphetamine (Zhu et al., 2006) and developmental injections of 3-nitropropionic acid (3-NP) cause a long-term loss of PV protein (Gibson and Clowry, 2003). Of particular interest is the vulnerability of cortical, hippocampal, and striatal PVIs to perinatal anoxia/hypoxia (Dell’Anna et al., 1996; Van de Berg et al., 2003; Gerstein et al., 2005; Wang et al., 2011) and global ischemia (Guan et al., 2000; Meade et al., 2000), considering that perinatal complications and/or hypoxia could be significant environmental factors contributing to the development of schizophrenia.

## PGC-1*α* as a molecular target of PVI-specific GxE

Recent evidence suggests that a critical regulator of antioxidant capacity in PVI could be the transcriptional coactivator peroxisome proliferator activated receptor gamma (PPARgamma) coactivator 1*α* (PGC-1*α*).

### PGC-1*α* drives developmental transcriptional programs for increased antioxidant capacity, parvalbumin transcription, and mitochondrial function

**Figure 2:**
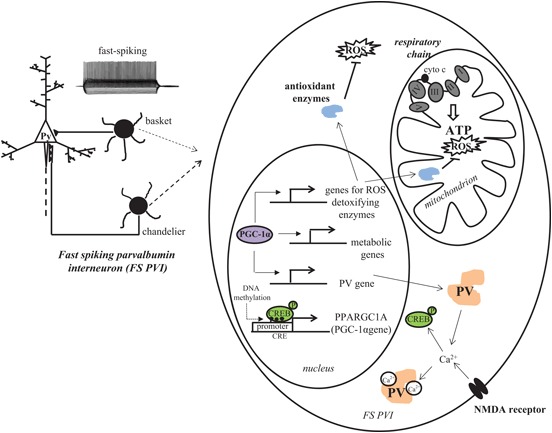
**PGC-1*α* is a critical contributor to the antioxidant capacity of FS PVIs.** The high energy demand of FS PVIs makes them vulnerable to mitochondrial dysfunction and oxidative stress. PGC-1*α*, a master regulator of transcriptional programs involved in mitochondrial energy metabolism and antioxidant defense, is highly concentrated in PVIs. In concert with other proteins, PGC-1*α* increases the transcription of antioxidant enzymes and PV. Transcription of PGC-1*α* itself is intracellular Ca^2+^ influx-dependent and can be regulated by epigenetic modifications.

PGC-1*α*, initially coined the “master regulator of metabolism,” is highly expressed in tissues with high energy demands, such as brown adipose tissue, heart, liver, skeletal muscle and brain (Puigserver et al., 1998). It has been found to serve as a central component of the transcriptional regulatory circuity that coordinately controls the energy-generating functions of mitochondria (Figure 2). It is capable of driving transcriptional control of mitochondrial biogenesis through direct interaction with, and coactivation of, PPARs (Madrazo and Kelly, 2008), estrogen-related receptors (ERRs; Schreiber et al., 2004; Eichner and Giguère, 2011), nuclear respiratory factors (NRF-1/NRF-2; Wu et al., 1999; Scarpulla, 2011) and the transcription factor yin-yang one (YY1; Basu et al., 1997; Seelan and Grossman, 1997; Cunningham et al., 2007; Xi et al., 2007), which are important nuclear transcription factors controlling mitochondrial metabolism (Scarpulla et al., 2012). PGC-1*α* is also an inducible responder to cellular energetic and metabolic stress, such as cold exposure (Puigserver et al., 1998; Uldry et al., 2006; Fisher et al., 2012), nutrient deprivation (Herzig et al., 2001; Yoon et al., 2001; Handschin et al., 2005; Rhee et al., 2006) and exercise (Baar et al., 2002; Handschin and Spiegelman, 2008) and is dynamically regulated in response to a variety of signaling pathways involved in cellular growth, differentiation and energy metabolism. Additionally, a large amount of evidence suggests that PGC-1*α* links mitochondrial biogenesis and the response to oxidative stress. PGC-1*α* has been shown to be a powerful regulator of ROS metabolism (St-Pierre et al., 2006; Cunningham et al., 2007).

In the central nervous system, PGC-1*α* protein is expressed most highly in GABAergic neurons (Cowell et al., 2007) with a particularly high concentration in cortical PVIs (Jiang et al., 2013). Reductions in PGC-1*α* mRNA or protein level and its activity have been implicated in several neurodegenerative diseases, such as Huntington (Cui et al., 2006; Weydt et al., 2006), Alzheimer’s (Qin et al., 2009), and Parkinson’s Disease (Zheng et al., 2010; Shin et al., 2011). A complete absence of PGC-1*α* in mice causes profound motor impairment and spongioform neurodegeneration in the striatum and cortex (Lin et al., 2004) by six weeks of age that is entirely attributable to a loss of PGC-1*α* in the central nervous system (Lucas et al., 2012). Of note, motor impairments are still observed in PGC-1*α* heterozygous mice, albeit to a lesser extent (Lucas et al., 2012); it is possible that the heterozygous mice would provide a better model to investigate the relevance of PGC-1*α* reductions for understanding its roles in pathophysiology.

The neuroprotective effect of endogenous PGC-1*α* has been explored in multiple different studies. Mice lacking PGC-1*α* are more sensitive to neurodegeneration induced by 1-methyl-4-phenyl-1, 2, 3, 6-tetrahydropyridine (MPTP) and kainic acid, and this degeneration was associated with greater levels of stable markers of oxidative damage, such as nitrotyrosylation of proteins and 8-OH-dG in DNA. Elevation of PGC-1*α* levels above those of wild-type nerve cells give an increased resistance to death by the oxidative stressors H_2_O_2_ and paraquat (St-Pierre et al., 2006), and overexpression of PGC-1*α* in culture increases mitochondrial density and ATP levels (Wareski et al., 2009), drives the expression of transcripts involved in antioxidant defense, glucose transport, and mitochondrial fusion and protects cells from hydrogen peroxide-induced cell death and caspase-3 activation (Cowell et al., 2009). In the context of NMDAR activation (Hardingham and Bading, 2010), knock-down of endogenous PGC-1*α* mediated by siRNA increases extrasynaptic NMDAR(EX) activity and vulnerability to excitotoxic insults in rat cortical neurons, while exogenous expression of PGC-1*α* cDNA results in a neuroprotective reduction of extrasynaptic currents without affecting synaptic NMDAR activity (Puddifoot et al., 2012).

Interestingly, PGC-1*α* protein is selectively localized to interneuron nuclei by the second week of postnatal life in rodent brain (Cowell et al., 2007), although its mRNA and protein are observed in neurons throughout the embryonic forebrain. This temporal pattern of expression coincides with the postnatal developmental switch of GABA from an excitatory to inhibitory neurotransmitter and the developmental induction of PV. In fact, mice lacking PGC-1*α* are deficient in PV-IR, without a loss of other interneuron markers such as GAD67, GAD65, calbindin, calretinin, cholescystokinin and somatostatin (Lucas et al., 2010). Changes in mRNA level of the PVI specific potassium channel Kv3.1 were not observed, either, suggesting that FS interneurons are intact in PGC-1*α* null mice, just lacking expression of PV protein. Furthermore, overexpression of PGC-1*α* cDNA in neuroblastoma cells robustly induces transcription of PV promoter, implicating PGC-1*α* as a critical factor for the developmental induction of PV gene transcription in cortical PVIs. In support of this hypothesis, we found substantial overlap of PV and PGC-1*α* mRNA in the cortex, with almost all PV mRNA-containing cells being PGC-1*α* mRNA-positive in S1 cortex and most of the total population of PGC-1*α* mRNA positive cells being PV mRNA-positive (Jiang et al., 2013). These observations raise the possibility that PGC-1*α* coordinately regulates programs of gene expression for the developmental induction of PV and an increased antioxidant capacity in PVIs (Figure 2).

With this in mind, we investigated whether PGC-1*α* mRNA or protein is affected by the developmental deletion of NMDARs in our Ppp1r2-cre/fGluN1 KO mice (Jiang et al., 2013). Cortical PGC-1*α* mRNA and protein levels were down-regulated and postnatal social isolation exacerbated the reduction. In addition to a reduction in PV-IR, mutant mice exhibited a reduction in mRNA levels of several key ROS-detoxifying enzymes including CuZnSOD, MnSOD, CAT and Gpx, and an impairment in antioxidant capacity and cortical oxidative stress. All these results suggest that PGC-1*α* plays a critical role in PVI antioxidant capacity that could have relevance for the understanding of schizophrenia pathophysiology. While it is currently not known whether PGC-1*α* expression is affected in schizophrenia, genetic association studies on chromosome 4p implicated the PGC-1*α* gene locus in bipolar disorder and schizophrenia (Blackwood et al., 1996; Christoforou et al., 2007, 2011). Integration of genetic data across human and murine studies also suggests PGC-1*α* as a potential susceptibility gene for anxiety-related disorders (Hettema et al., 2011).

Concerning the potential roles for PGC-1*α* in the maintenance of mitochondrial function, there is substantial evidence for mitochondrial dysfunction in schizophrenia. Using ^31^phosphorus magnetic resonance spectroscopy (^31^P-MRS) to measure ATP and phospholipids (Fujimoto et al., 1992; Volz et al., 1997), and positron emission tomography (PET) scans with [^18^F]-fluoro-deoxy-glucose (FDG; Buchsbaum and Hazlett, 1998), altered energy metabolism has been observed in cerebral cortex of schizophrenic patients. A significant decrease in mitochondria number and density in the prefrontal cortex and caudate nucleus of postmortem brains of subjects with schizophrenia was observed compared with control subjects (Uranova et al., 2001). A lower number of mitochondria were found in medication-free patients compared with those taking antipsychotics or control medications, suggesting drug treatment normalizes the number of mitochondria (Inuwa et al., 2005). Combining a parallel transcriptomics, proteomics and metabomics approach, Prabakaran et al. (2004) explored the molecular signatures in brain tissue of schizophrenia. Almost half of the altered proteins identified were associated with mitochondrial function and oxidative stress responses. Critical role of PGC-1*α* in mitochondrial biogenesis and metabolism highlights it as an interesting candidate for further study in the context of schizophrenia.

### Potential biological mechanisms underlying convergence of GxE on PGC-1*α*

It is notable in our study that no significant change in PGC-1*α* mRNA or protein level in Ppp1r2-cre/fGluN1 KO mice was observed until the post-adolescent period (eight week old). Post-weaning social isolation exacerbated the reduction of PGC-1*α* only in KO mice. This implicates that PGC-1*α*, as a master regulator of mitochondria energy metabolism and anti-oxidation, with a selective expression pattern in FS PVI, could be a potential converging factor for GxE. However, the mechanisms by which GxE regulate PGC-1*α* transcription and trigger oxidative stress on PVIs remains to be clarified.

#### Early postnatal NMDAR hypofunction in cortical PVI

Notably, PGC-1*α* transcription and activity itself can be regulated by alterations in intracellular calcium concentrations and the activation of calcium calmodulin-dependent kinase IV (Handschin et al., 2003) that often occur with activation of the NMDAR. In fact, in neurons, transcription of PGC-1*α* is activity-dependent (Liang and Wong-Riley, 2006; Meng et al., 2007; Yu and Yang, 2010) and increased by NMDAR activation (Lee et al., 2007; Luo et al., 2009; Liang et al., 2010; Figure 2). Thus, it is not surprising that early postnatal KO of NMDAR in PVIs is associated with a down-regulation of PGC-1*α*.

#### Epigenetic regulation of PGC-1*α*

Another mechanism of PGC-1*α* regulation that could account for environmental and genetic convergence on PVIs may be the modulation of PGC-1*α* transcription and/or activity by epigenetic mechanisms. Increasing evidence suggests that external environmental factors, such as nutritional, chemical, physical, even psychosocial factors can modify gene expression through epigenetic processes (Jirtle and Skinner, 2007). Epigenetics refers to a set of mitotically heritable and reversible changes in gene expression that occur without a change in the genomic DNA sequence. Epigenetic changes have been associated with a number of paradigms involving social behavior in animal models, such as maternal care (Weaver et al., 2004), early life adversity (Murgatroyd et al., 2009), animal models of posttraumatic stress disorder (PTSD; Chertkow-Deutsher et al., 2010) and chronic social defeat (Tsankova et al., 2006).

Furthermore, epigenetic changes in interneuron-specific transcripts have been documented in schizophrenia; a significant reduction of GABAergic protein (GAD67 and reelin) are accompanied by increased methylation of the GAD67 and Reelin promoters and increased DNA methyltransferase (DNMT) one in the same interneurons in the cortex of schizophrenic patients (Guidotti et al., 2000; Grayson et al., 2005; Veldic et al., 2005; Huang et al., 2007; Ruzicka et al., 2007). Mill et al. (2008) published the first epigenome-wide study of psychosis using postmortem tissue obtained from frontal cortex of patients with schizophrenia or bipolar disorders. This study enriched the unmethylated fraction of genome DNA and used a CpG island microarray to assay DNA methylation at approximately 12,000 sites across the genome. Their gene-ontology analysis highlights epigenetic disruption to loci involved in mitochondrial function, stress response and brain development.

Recently, another study utilizing genome-wide methylation profiling of blood samples from monozygotic twins discordant for schizophrenia linked aberrant DNA methylation of PGC-1*α* gene to schizophrenia (Dempster et al., 2011). Indeed, one CRE (CREB binding site) was found in mouse PGC-1*α* promoter region and it encompassed a CpG site (Figure 2). It has been reported that methylation of CpG in CRE blocked pCREB binding (Iguchi-Ariga and Schaffner, 1989; Sunahori et al., 2009). CREB is an important regulator for PGC-1*α* gene transcription (Herzig et al., 2001; St-Pierre et al., 2006). Barrès et al. (2009) provided evidence that PGC-1*α* hypermethylation is concomitant with reduced mitochondrial content in type 2 diabetic patients, and links DNMT3B to the acute fatty-acid-induced non-CpG methylation of PGC-1*α* promoter. Although studies by Dempster et al. (2011) and others assessed PGC-1*α* methylation status in peripheral samples, and the methylation response in peripheral cells may not accurately reflect methylation status in the brain (Provençal et al., 2012), these data suggest that a differential epigenetic response does occur on PGC-1*α* in schizophrenic individuals compared with normal subjects. There is also evidence that PGC-1*α* gene expression and activity are influenced directly by inhibitors of histone deacetylases (HDACs), enzymes that epigenetically modify histones to block transcription. Overexpression of HDAC5 reduces PGC-1*α* in the heart (Czubryt et al., 2003) by blocking the developmental induction of PGC-1*α* by myocyte enhancing factor 2 (MEF2), and exposure of neuroblastoma cells to the HDAC inhibitors trichostatin A or valproic acid (a mood stabilizer given as adjunct therapy to schizophrenia patients) increases mRNA level of PGC-1*α* and its target gene glucose transporter four (Cowell et al., 2009). Hypoxia exposure itself can cause an increase in the expression of the DNA methylating enzymes DNMT1 and DNMT3b mRNA that is sustained in adulthood and accompanied by increased methylation of the SOD2 promoter (Nanduri et al., 2012); taking into account the role of PGC-1*α* in tissue responses to hypoxia (Arany et al., 2008; Zhu et al., 2010; Pino et al., 2012; Zhao et al., 2012), it is attractive to speculate that environmental stimuli can modulate PGC-1*α*-dependent pathways and PVI antioxidant capacity by epigenetic mechanisms.

Hypothalamic-pituitary-adrenocortical (HPA) hyperactivity by social isolation/stress could involve into PGC-1*α* regulation through epigenetic mechanisms as well. Activation of the HPA axis represents a common reaction to many environmental insults, including infection, malnutrition, hypoxia, and psychosocial stress (Oitzl et al., 2010). Most of rodent studies showed that chronic early life social isolation is associated with higher basal levels of glucocorticoids and increased HPA response to acute stressors (Weiss et al., 2004; Chida et al., 2005; Perelló et al., 2006; Ruscio et al., 2007; Williams et al., 2009; Weintraub et al., 2010; Toth et al., 2011). Glucocorticoids have been reported to influence DNA methylation in genes containing glucocorticoid response elements (GREs), such as fkbp5 and tyrosine hydroxylase gene (Lee et al., 2011; Niwa et al., 2013). Studies in early postnatal maternal care/separation have linked epigenetic mechanisms with alteration of genes involved in the regulation of the HPA axis. Early life experience influences DNA methylation state of glucocorticoid receptor (GR) gene in human studies (Oberlander et al., 2008; McGowan et al., 2009; Labonte et al., 2012) and animal models (see review by, Weaver, 2009). Epigenetic-mediated changes in GR could contribute to PGC-1*α* alteration. Further studies need to be done to clarify how GxE influence the transcription of PGC-1*α* and PV and antioxidant capacity in cortical PVIs.

## Summary

The interaction between genes and environment (GxE) has been postulated to be critical to trigger the onset of schizophrenia. Epidemiological observations and neuropathological studies all support that schizophrenia is a consequence of abnormal brain development. As an essential player in the generation of gamma oscillations, corticolimbic PVIs have a key role in higher cognitive processing. Considering the evidence for PVI dysfunction in patients with schizophrenia and in various neurodevelopmental animal models, the vulnerability of PVIs during early life development and the mechanisms by which these neurons respond to environmental stimuli are particularly relevant for our understanding of schizophrenia pathophysiology. Here, we presented evidence for a convergence of genetic and environmental factors on oxidative stress in PVIs and suggest that PGC-1*α* is a critical regulator of PV transcription and antioxidant capacity in these neurons. The delayed maturation during postnatal period and high energy demanding of FS PVIs make them susceptible to environmental insults, particularly redox imbalance, during early life experience. Oxidative stress could be one of the important mechanisms integrates GxE on PVIs disturbance. PGC-1*α*, as a master regulator of mitochondrial metabolism, which is highly and selectively expressed in PVIs, could be one of the key factors responsible for the antioxidant capacity of PVIs. Our study using NMDAR hypofunction animal model revealed that redox dysregulation due to a decrease in PGC-1*α* abundance in cortical parvalbumin interneurons exacerbated schizophrenia-like phenotypes following social isolation stress. This implicates PGC-1*α* as a potential converging factor for GxE. Further studies on the impact of GxE on PGC-1*α* transcription and function would not only help identify neuropathophysiological mechanism underlying GxE in schizophrenia, but importantly, aid in the development of targeted interventions for this illness.

## Conflict of interest statement

The authors declare that the research was conducted in the absence of any commercial or financial relationships that could be construed as a potential conflict of interest.
